# Clinical significance and prognostic value of receptor conversion in hormone receptor positive breast cancers after neoadjuvant chemotherapy

**DOI:** 10.1186/s12957-018-1332-7

**Published:** 2018-03-07

**Authors:** Libo Yang, Xiaorong Zhong, Tianjie Pu, Yan Qiu, Feng Ye, Hong Bu

**Affiliations:** 1 Department of Pathology, West China Hospital, Sichuan University, Chengdu, Sichuan Province China; 2 Departments of Head and Neck and Mammary Gland Oncology, and Medical Oncology, Cancer Center, West China Hospital, Sichuan University, Chengdu, Sichuan Province China; 3 Laboratory of Pathology, West China Hospital, Sichuan University, Guo Xue Xiang No.37, Chengdu, Sichuan Province 610041 China

**Keywords:** Breast cancer, Neoadjuvant chemotherapy, Hormone receptor, Receptor conversion

## Abstract

**Background:**

Neoadjuvant chemotherapy (NAC) is widely used in advanced breast cancer patients. However, there is little known about conversion frequency of estrogen receptor (ER) and/or progesterone receptor (PR) status for hormone receptor positive-breast cancer patients after NAC and their correlation with prognosis.

**Methods:**

In this study, 231 breast cancer patients with residual disease after NAC were enrolled and divided into receptor stable group (having no conversion in both ER and PR status pre- and post-NAC) and any receptor conversion group (having any conversion in either ER or PR status). Univariate and multivariate survival analyses were used to compare survival differences between the two groups.

**Results:**

Fifty-five patients (23.8%) had ER and/or PR conversion after NAC. Younger patients (≤ 50 years) were more likely to have receptor conversion (*P* = 0.014). For 213 patients (92.2%) who received adjuvant endocrinotherapy after surgery, the 5-year disease free survival (DFS) estimates for patients in the any receptor conversion group (55.2%) was worse than patients in the receptor stable group (73.7%, Log-rank test, *P* = 0.015). While the 5-year overall survival estimates for patients with or without receptor conversion were not statistically different (86.0 vs. 82.4%, Log-rank test, *P* = 0.587). In multivariate Cox proportional hazard analyses, patients with any receptor conversion had worse DFS (hazard ratio, 1.995; 95% confidence interval, 1.130–3.521, *P* = 0.031).

**Conclusions:**

It is necessary to recommend patients to test biomarkers in residual disease and pay more attention to patients who have any receptor conversion. These patients may need more individual therapy after surgery.

## Background

Neoadjuvant chemotherapy (NAC) has been generally accepted in patients with locally advanced breast cancer. Besides reducing tumor stage, NAC is a practical approach to individually test chemosensitivity preoperatively. And some patients who reached pathologic complete response (pCR) through NAC tend to have prefer prognosis compared with those who still have residual disease [1–4]. Guarneri et al. [2] reported that 5-year disease-free survival rates were 87.1 vs. 61.1% and 10-year disease-free survival rates were 74.9 vs. 39.9% for patients with and without pCR, respectively.

Before treatment, core needle biopsy (CNB) is essential for diagnosis and biomarkers assessment. Hormone (estrogen and progesterone) receptors (HR) are important biomarkers in breast cancers and indication of adjuvant endocrinotherapy [5–7]. However, patients with HR expression tumors benefit less from chemotherapy since they have lower pCR rates compared with patients with HR negative [8–10]. Conversely, HR expression is a predictor of favorable prognosis in breast cancers partially because of adjuvant endocrinotherapy. In general, HR status has an unreplaceable predictive and prognostic value for breast cancer patients.

Immunohistochemistry (IHC) is a standard method to quantify HR expression mainly based on the fraction of stained tumor cells. Several studies have reported conversions of estrogen receptor (ER) and progesterone receptor (PR) status between CNB and surgical specimens after NAC [11–13]. Conversions of receptor are also prevalent in patients that did not receive NAC, it is statistically more frequent in NAC patients [14]. However, the influences of these conversions on following treatment and prognosis in breast cancers are still unclear.

The objectives of this study were to assess the conversion frequency of ER and/or PR status in HR expression patients after NAC and to determine whether these conversions influence prognosis.

## Methods

### Patients and samples

This study was approved by the institutional Ethics Committee of the West China Hospital. We retrospectively collected the clinicopathological data of 256 breast cancer patients with HR expression (ER and/or PR positive) in pre-NAC specimens between January 2009 and December 2012. All those patients were in clinical tumor stage II to III with a follow-up time more than 6 months. In this study, the regimen of NAC was based on anthracycline and/or taxane. No patient received adjuvant anti-HER2-targeted therapy or endocrine therapy before surgery. Before NAC, patients underwent ultrasound-guided needle biopsy for histological examination. Pathologists at West China Hospital reconfirmed hematoxylin and eosin (H&E) slides of specimens and evaluated residual tumor cells at primary tumor site (breast) of surgical specimens. Histological type was also recorded for analyses.

**Fig. 1:**
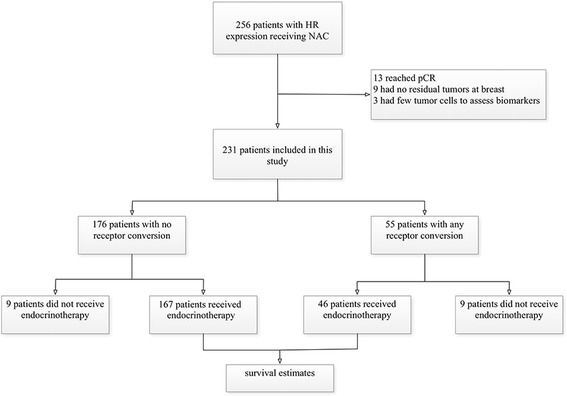
Flowchart showing the inclusion of this study. (HR, hormone receptor; pCR, pathologic complete response)

Therefore, 25 patients were excluded from this study because of no or few tumor cells in primary tumor site. Finally, 231 formalin-fixed paraffin-embedded (FFPE) pre-NAC and matched surgical specimens were included in this study (Fig. 1).

### Immunohistochemistry

Biomarker status were evaluated using pre-NAC and surgical FFPE tissue blocks by IHC. IHC staining of tumor tissues for ER (Confirm anti-ER (SP1), rabbit monoclonal antibody, Ventana Medical Systems), PR (Confirm anti-PR (1E2), rabbit monoclonal antibody, Ventana Medical Systems), human epidermal growth factor receptor 2 (HER2) (Ventana anti-HER2/neu (4B5), rabbit monoclonal antibody, Ventana Medical Systems), and Ki67 (Confirm anti-Ki67 (30–9), rabbit monoclonal antibody, Ventana Medical Systems) was performed using the automated Benchmark XT platform (Ventana Medical Systems) and according to the manufacturer’s recommendations. The cutoff value of ER- and PR-positive disease was no less than 1% positively stained nuclei in tumor tissues. HER2-positive status was defined as 3(+) by IHC or amplification confirmed by fluorescence in situ hybridization (FISH). Ki67 expression was divided into high expression group (≥ 15%) and low expression group (< 15%).

### Statistical analysis

In this study, patients with residual disease at primary tumor site after NAC were classified as receptor stable group (having no conversion in both ER and PR status) and any receptor conversion group (having any conversion in either ER or PR status). And in this study, receptor conversion meant receptor status changed from positive to negative or from negative to positive after NAC. Pearson *χ*^*2*^ test was used to compare pre-NAC clinicopathological differences between the two groups. Patient prognosis was evaluated through disease-free survival (DFS) and overall survival (OS). DFS was defined as the interval from the date of surgery to the detection of relapse, death from any cause, or the date of the last visit for patients without events. OS was defined as the interval from the date of surgery to death or the date of the last visit for patients without events. Survival analyses were performed using Kaplan-Meier method for DFS and OS. The survival curves were compared using Log-rank test. Univariate and multivariate Cox proportional hazard analyses were used to determine the association of clinicopathological factors with DFS and OS. The two-sided significance level was set at *P* <  0.05. In addition, multivariate Cox proportional hazard analyses only included variables that were statistically significant (*P* <  0.05) in univariate analyses.

## Results

### Patient characteristics

Twenty-two of 256 HR positive patients had no residual tumor cell at primary tumor site and 13 patients reached pCR through NAC. The pCR rate in those patients was 5.08%. Three patients that had few residual tumor cells at the primary tumor site that were inadequate for biomarker assessment were also excluded from study. For the remaining 231 patients, 158 patients (68.4%) were under 50 years old, and the mean age of all patients was 46.41 ± 8.93 years. About tumor histological type, 201 patients (87.0%) had invasive ductal carcinoma, 17 patients (7.4%) had invasive ductal carcinoma mixed with other invasive carcinoma, and 13 patients (5.6%) had other invasive carcinoma, such as invasive lobular carcinoma (7 patients), mucinous adenocarcinoma (5 patients), and invasive cribriform carcinoma (1 patients). Before receiving NAC, 41 patients (17.7%) had tumor invading chest wall and 199 patients (86.1%) had lymph node involvement. Though all included tumors were HR positive (ER and/or PR positive), 221 tumors (95.7%) were ER positive and 200 tumors (86.6%) were PR positive. HER2 status was positive in 37 patients (16.0%), and Ki67 expression was high in 172 patients (74.5%). All patients underwent one to eight of NAC using anthracycline-based regimens (30.7%), anthracycline- and taxane-based regimens (60.6%), and taxane-based regimens (8.7%).

### Conversion in receptor status

**Fig. 2:**
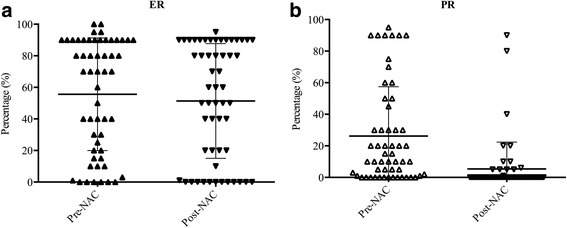
Percentage of ER and PR expression pre- and post-NAC for patients with any receptor conversion. **a** Percentage of ER expression pre- and post-NAC for patients with any receptor conversion. **b** Percentage of PR expression pre- and post-NAC for patients with any receptor conversion. (ER estrogen receptor, PR progesterone, NAC neoadjuvant chemotherapy)

Patients’ characteristics before NAC according to receptor conversion are listed in Table 1. Fifty-five patients (23.8%) had ER and/or PR conversion after NAC. The percentage of ER and PR expression pre- and post-NAC was shown in Fig. 2. ER conversion happened in 13 patients (5.6%), which contained positive-to-negative conversion in 9 patients and negative-to-positive conversion in 4 patients. Meanwhile, PR conversion happened in 45 patients (19.5%), which contained positive-to-negative conversion in 37 patients and negative-to-positive conversion in 8 patients. Moreover, 11 patients (4.8%) had HR negative (ER and PR negative) disease after NAC. We found younger (≤ 50 years) patients tended to convert in receptor status more frequently (*P* = 0.014). However, there were no significant differences in tumor stage, node status, nuclear grade, histology, NAC regimens or cycles, HER2 status, and Ki-67 status between the receptor stable group and the any receptor conversion group.

**Table 1:** Patients’ characteristics according to receptor stable and any receptor conversion

Characteristics	All patients	Receptor stable	Any receptor conversion	*P*
	(*N* = 231)	(*N* = 176)	(*N* = 55)	
Age, years				
≤ 50	158 (68.4)	113 (64.2)	45 (81.8)	0.014
> 50	73 (31.6)	63 (35.8)	10 (18.2)	
Initial tumor stage				
T1	41 (17.7)	34 (19.3)	7 (12.7)	0.500
T2	119 (51.5)	90 (51.1)	29 (52.7)	
T3 or T4	71 (30.7)	52 (29.5)	19 (34.5)	
Initial node status				
Negative	32 (13.9)	22 (12.5)	10 (18.2)	0.287
Positive	199 (86.1)	154 (87.5)	45 (81.8)	
Nuclear grade				
1 or 2	74 (32.0)	60 (56.6)	14 (46.7)	0.335
3	62 (26.8)	46 (43.4)	16 (53.3)	
NA	95 (41.1)	–	–	
Histology				
IDC	201 (87.0)	154 (87.5)	47 (85.5)	0.832
Mixed	17 (7.4)	13 (7.4)	4 (7.3)	
Other	13 (5.6)	9 (5.1)	4 (7.3)	
Initial ER status				
Negative	10 (4.3)	3 (1.7)	7 (12.7)	< 0.001
Positive	221 (95.7)	173 (98.3)	48 (87.3)	
Initial PR status				
Negative	31 (13.4)	18 (10.2)	13 (23.6)	0.011
Positive	200 (86.6)	158 (89.8)	42 (76.4)	
Initial HER2 status				
Negative	194 (84.0)	150 (85.2)	44 (80.0)	0.356
Positive	37 (16.0)	26 (14.8)	11 (20.0)	
Initial Ki-67 status				
< 15%	59 (25.5)	48 (27.3)	11 (20.0)	0.280
≥ 15%	172 (74.5)	128 (72.7)	44 (80.0)	
NAC cycles				
1–4	153 (66.2)	117 (66.5)	36 (65.5)	0.889
5–8	78 (33.8)	59 (33.5)	19 (34.5)	
NAC regimens				
Anthracycline-based	71 (30.7)	53 (30.1)	18 (32.7)	0.879
Anthracycline- and taxane-based	140 (60.6)	107 (60.8)	33 (60.0)	
Taxane-based	20 (8.7)	16 (9.1)	4 (7.3)	

*ER* estrogen receptor, *PR* progesterone receptor, *HER2* human epidermal growth factor receptor 2, *NAC* neoadjuvant chemotherapy

### Follow-up and survival analysis

Adjuvant endocrinotherapy was given to 167 (94.9%) of 176 patients in the receptor stable group and 46 (83.6%) of 55 patients in the any receptor conversion group. For patients who had HR negative disease after NAC, there were 45.5% (5 of 11) of patients who still received adjuvant endocrinotherapy depending on personal and/or oncologist preferences. In total, there were 18 patients who did not receive adjuvant endocrinotherapy after surgery. And those patients were not included into the next survival analyses.

**Fig. 3:**
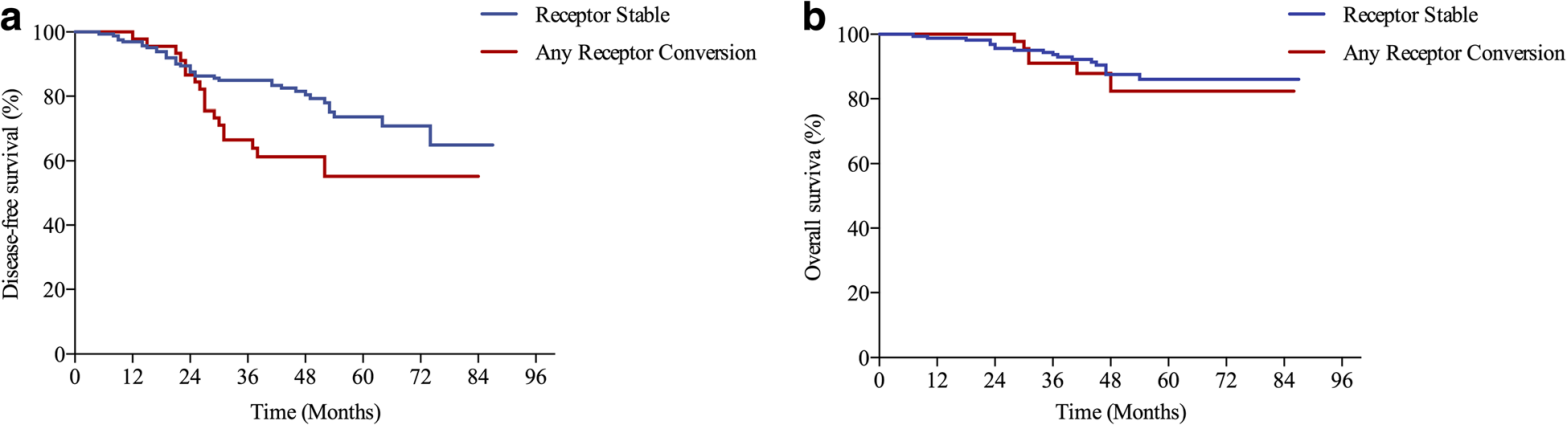
Survival estimates according to receptor conversion status. **a** Disease-free survival estimates (Log-rank test, *P* = 0.015). **b** Overall survival estimates (Log-rank test, *P* = 0.587)

During the follow-up time (range, 7–87 months), 24 (11.3%) of 213 patients had died, and 54 (25.4%) of 213 patients had experienced disease recurrence. The 5-year DFS estimates for patients in the receptor stable group (73.7%) were significantly higher than patients in the any receptor conversion group (55.2%, *P* = 0.015, Fig. 3a); however, the 5-year OS estimates between patients in the receptor stable group (86.0%) and patients in the any receptor conversion group (82.4%) were not significantly different (*P* = 0.587, Fig. 3b).

The univariate and multivariate Cox regression analyses of DFS and OS about pre- and post-NAC characteristics in 213 patients who received adjuvant endocrinotherapy are listed in Tables 2 and 3. In this study, patients were divided into the receptor stable group and the no receptor conversion group according to pre- and post-NAC ER and PR status. Therefore, pre- and post-NAC ER and PR status were not included in the survival analyses to avoid interaction effect with patient grouping. Moreover, 7 (22.6%) of 31 tumors had positive-to-negative change, and 5 (2.7%) of 182 tumors had negative-to-positive change in HER2 status for patients included in the survival analyses. So, both pre- and post-NAC HER2 status were considered in the analyses. The multivariate Cox proportional hazard analyses only included variables that were statistically significant (*P* < 0.05) in univariate analyses. Any receptor conversion status was a significant predictor of DFS (hazard ratio (HR), 1.995; 95% confidence interval (CI), 1.130–3.521; *P* = 0.031).

**Table 2:** Univariate and multivariate analyses of disease-free survival for initial HR-positive patients with adjuvant endocrinotherapy after surgery

Characteristics	*N* (%)	DFS			
		Uv		Mv	
		*P*	HR (95% CI)	*P*	HR (95% CI)
Age, years		0.181			
≤ 50	146 (68.5)		1.00		
> 50	67 (31.5)		0.645 (0.340–1.226)		
Initial tumor stage		0.649			
T1	38 (17.8)		1.00		
T2	107 (50.2)	0.364	1.436 (0.658–3.136)		
T3 or T4	68 (31.9)	0.428	1.413 (0.601–3.321)		
Initial node status		0.037		0.481	
Negative	26 (12.2)		1.00		1.00
Positive	187 (87.8)		4.506 (1.093–18.570)		1.839 (0.338–9.992)
Nuclear grade		0.246			
1 or 2	73 (34.3)		1.00		
3	55 (25.8)		1.475 (0.765–2.845)		
NA	85 (39.9)	–	–		
Histology		0.877			
IDC	183 (85.9)		1.00		
Mixed	17 (8.0)	0.802	1.126 (0.446–2.841)		
Other	13 (6.1)	0.639	1.277 (0.459–3.554)		
Initial HER2 status		0.618			
Negative	182 (85.4)		1.00		
Positive	31 (14.5)		1.200 (0.586–2.456)		
Initial Ki-67 status		0.584			
< 15%	57 (26.8)		1.00		
≥ 15%	156 (73.2)		1.191 (0.638–2.223)		
NAC regimens		0.058			
Anthracycline-based	65 (30.5)		1.00		
Anthracycline- and Taxane-based	133 (62.4)	0.895	0.959 (0.512–1.797)		
Taxane-based	15 (7.0)	0.043	2.561 (1.032–6.357)		
Any receptor conversion		0.017		0.031	
No	167 (78.4)		1.00		1.00
Yes	46 (21.6)		1.995 (1.130–3.521)		1.881 (1.058–3.342)
Tumor size at surgery		0.694			
≤ 2 cm	88 (41.3)		1.00		
> 2 and ≤ 5 cm	114 (53.5)	0.393	1.282 (0.725–2.267)		
> 5 cm	11 (5.2)	0.806	1.165 (0.344–3.944)		
Node status at surgery		0.005		0.095	
Negative	49 (23.0)		1.00		1.00
Positive	164 (77.0)		3.774 (1.498–9.507)		2.556 (0.846–7.698)
HER2 status at surgery		0.587			
Negative	184 (86.4)		1.00		
Positive	29 (13.6)		0.790 (0.338–1.848)		
Ki-67 status at surgery		0.405			
< 15%	116 (54.5)		1.00		
≥ 15%	97 (45.5)		1.255 (0.736–2.142)		
Radiotherapy		0.031		0.329	
No	57 (26.8)		1.00		1.00
Yes	156 (73.2)		2.280 (1.076–4.831)		1.476 (0.675–3.227)

*DFS* disease-free survival, *HR* hazard ratio, *CI* confidence interval, *Uv* univariate Cox proportional hazard analyses, *Mv* multivariate Cox proportional hazard analyses, *IDC* invasive ductal carcinoma, *HER2* human epidermal growth factor receptor 2, *NAC* neoadjuvant chemotherapy

**Table 3:** Univariate and multivariate analyses of overall survival for initial HR-positive patients with adjuvant endocrinotherapy after surgery

Characteristics	*N* (%)	OS			
		Uv		Mv	
		*P*	HR (95% CI)	*P*	HR (95% CI)
Age, years		0.089			
≤ 50	146 (68.5)		1.00		
> 50	67 (31.5)		2.007 (0.899–4.482)		
Initial tumor stage		0.073			
T1	38 (17.8)		1.00		
T2	107 (50.2)	0.757	1.230 (0.333–4.457)		
T3 or T4	68 (31.9)	0.094	2.967 (0.832–10.579)		
Initial node status		0.242			
Negative	26 (12.2)		1.00		
Positive	187 (87.8)		3.302 (0.446–24.467)		
Nuclear grade		0.335			
1 or 2	73 (34.3)		1.00		
3	55 (25.8)		1.651 (0.596–4.577)		
NA	85 (39.9)				
Histology		0.042		0.214	
IDC	183 (85.9)		1.00		1.00
Mixed	17 (8.0)	0.19	3.334 (1.221–9.104)	0.154	2.196 (0.745–6.469)
Other	13 (6.1)	0.177	2.339 (0.681–8.039)	0.208	2.228 (0.641–7.740)
Initial HER2 status		0.417			
Negative	182 (85.4)		1.00		
Positive	31 (14.5)		0.549 (0.129–2.337)		
Initial Ki-67 status		0.128			
< 15%	57 (26.8)		1.00		
≥ 15%	156 (73.2)		0.532 (0.236–1.199)		
NAC regimens		0.002		0.015	
Anthracycline-based	65 (30.5)		1.00		1.00
Anthracycline- and Taxane-based	133 (62.4)	0.574	1.378 (0.451–4.208)	0.642	1.307 (0.424–4.031)
Taxane-based	15 (7.0)	0.003	6.712 (1.893–23.794)	0.013	5.242 (1.408–19.509)
Any receptor conversion		0.588			
No	167 (78.4)		1.00		
Yes	46 (21.6)		1.291 (0.512–3.259)		
Tumor size at surgery		0.223			
≤ 2 cm	88 (41.3)		1.00		
> 2 and ≤ 5 cm	114 (53.5)	0.313	1.596 (0.644–3.957)		
> 5 cm	11 (5.2)	0.087	3.263 (0.843–12.633)		
Node status at surgery		0.071			
Negative	49 (23.0)		1.00		
Positive	164 (77.0)		3.802 (0.893–16.182)		
HER2 status at surgery		0.976			
Negative	184 (86.4)		1.00		
Positive	29 (13.6)		0.982 (0.292–3.294)		
Ki-67 status at surgery		0.676			
< 15%	116 (54.5)		1.00		
≥ 15%	97 (45.5)		1.187 (0.531–2.654)		
Radiotherapy		0.859			
No	57 (26.8)		1.00		
Yes	156 (73.2)		1.088 (0.432–2.740)		

*OS* overall survival, *HR* hazard ratio, *CI* confidence interval, *Uv* Univariate Cox proportional hazard analyses, *Mv* multivariate Cox proportional hazard analyses, *IDC* invasive ductal carcinoma, *HER2* human epidermal growth factor receptor 2, *NAC* neoadjuvant chemotherapy

## Discussion

This study focused on ER and/or PR conversion after receiving NAC and its influences on the prognosis and adjuvant endocrinotherapy in breast cancer patients. Only HR positive tumors are recommended to receive adjuvant endocrinotherapy. There are no clear guidelines about adjuvant endocrinotherapy for those patients who had ER and/or PR conversion after NAC. Therefore, only HR-positive disease patients before NAC were enrolled to reduce the influence of adjuvant endocrinotherapy after surgery. In addition, patients with HR negative disease were more sensitive to chemotherapy [15, 16], and less patients had residual disease at breast after NAC to assess biomarkers.

In this retrospective study, we set positive ER and PR expression cutoff value as 1%. For 231 HR-positive disease before NAC, 55 (23.8%) had ER and/or PR conversion, 13 (5.6%) had ER conversion, and 45 (19.5%) had PR conversion. Hirata et al. [12] used 10% as the cutoff value for ER and PR expression. Of 214 HR positive disease in their study, 38 (17.8%) had ER conversion and 89 (41.6%) had PR conversion after NAC. Other studies which used a cutoff of 5% or Allred score (≥ 3) to define ER and PR positive staining also reported discordance of receptor expression after NAC [11, 17]. Except for sampling error of core needle biopsy before treatment, receiving NAC may be a main cause of this phenomenon [14]. Our analysis show that younger (≤ 50 years) patients were more likely to have conversion, due to an unknown underlying mechanism.

The influence of HR status changes to prognosis remained controversial. Hirata et al. [12] did not find a significant difference in DFS and OS between the HR-positive group and HR-conversion group. However, in their study, there were 18 (38.3%) of 47 patients who had HR negative tumor before NAC in the HR conversion group. Chen et al. [18] adjusted the influence of adjuvant endocrinotherapy in HR positive patients and found negative influence in DFS and OS for patients who had HR positive to negative change after NAC. Parinyanitikul et al. [19] used the 20% cutoff for absolute percent change for ER and PR expression and found that patients with any receptor change had better relapse-free survival (RFS) than patients with no receptor change. Those previous studies focused on the influence of HR status conversion or absolute percent change of ER or PR expression, but there were few studies focused on the influences of at least one of ER and PR status conversion in HR positive patients. After adjusting the influence of adjuvant endocrinotherapy, patients with receptor stable disease had significantly higher DFS than patients with any receptor conversion (*P* = 0.017). Our multivariable analysis also revealed that any receptor conversion status was associated with worse DFS (HR, 1.881; 95% CI, 1.058–3.342; *P* = 0.031). While 5-year OS estimates for patients without any receptor conversion was higher than the other patients (86.0 vs. 82.4%), the difference was not statistically significant in univariate analysis (*P* = 0.588). Moreover, we also analyzed survival differences for patients who did not receive endocrinotherapy between the two groups. For nine patients with any receptor conversion, the 5-year DFS and 5-year OS rates were 77.8 and 88.9%, respectively. And these rates were not only similar to patients with no receptor conversion (88.9 and 88.9%) but also similar to patients with any receptor conversion who received adjuvant endocrinotherapy (60.8 and 87.0%).

In this study, the proportion of HER2 positive-to-negative change was higher than negative-to-positive change (22.6 vs. 2.7%). However, it only reflected change pattern of tumors which were HR-positive before NAC. Niikura et al. [20] reported change pattern of HER2 status regardless of pre-NAC HR status, and they had similar results (21.4% for HER2 positive-to-negative change and 3.4% for HER2 negative-to-positive change) to ours. Though HER2 status may have conversion after receiving NAC, this phenomenon was not significant when compared with patients who did not receive NAC [14]. By the way, 52.4% of patients had low Ki-67 expression after NAC, and it was much more than the proportion (25.5%) before NAC (*P* < 0.01). Above 60% of breast cancer patients with non-pCR disease had reduction of Ki-67 expression after NAC [21, 22]. Some studies had also reported that patients with reduction of Ki-67 expression had more favorable prognosis after NAC [21, 22]. In our present data, we did not do further study on the influence of reduction of Ki-67 expression on prognosis.

## Conclusions

Our study showed that conversion in ER and/or PR status after NAC should not be neglected for breast cancer patients. Patients with any conversion in ER or PR status was associated with poor DFS after adjusting for adjuvant endocrinotherapy. Biomarkers testing is recommended in patients with residual disease for receptor conversion. These patients may need more individualized therapy after surgery.

## Abbreviations

CNB: Core needle biopsy
DFS: Disease-free survival
ER: Estrogen receptor
HER2: Human epidermal growth factor receptor 2
HR: Hormone receptor
IHC: Immunohistochemistry
NAC: Neoadjuvant chemotherapy
OS: Overall survival
pCR: Pathologic complete response
PR: Progesterone receptor

## References

[1] Rastogi P, Anderson SJ, Bear HD (2008). Preoperative chemotherapy: updates of National Surgical Adjuvant Breast and Bowel Project Protocols B-18 and B-27. J Clin Oncol.

[2] Guarneri V, Broglio K, Kau SW (2006). Prognostic value of pathologic complete response after primary chemotherapy in relation to hormone receptor status and other factors. J Clin Oncol.

[3] Kuerer HM, Newman LA, Smith TL (1999). Clinical course of breast cancer patients with complete pathologic primary tumor and axillary lymph node response to doxorubicin-based neoadjuvant chemotherapy. J Clin Oncol.

[4] Liedtke C, Mazouni C, Hess KR (2008). Response to neoadjuvant therapy and long-term survival in patients with triple-negative breast cancer. J Clin Oncol.

[5] Early Breast Cancer Trialists’ Collaborative Group (1998). Tamoxifen for early breast cancer: an overview of the randomised trials. Lancet.

[6] Early Breast Cancer Trialists’ Collaborative Group (2005). Effects of chemotherapy and hormonal therapy for early breast cancer on recurrence and 15-year survival: an overview of the randomised trials. Lancet.

[7] Davies C, Godwin J, Early Breast Cancer Trialists’ Collaborative Group (2011). Relevance of breast cancer hormone receptors and other factors to the efficacy of adjuvant tamoxifen: patient-level meta-analysis of randomised trials. Lancet.

[8] Li XR, Liu M, Zhang YJ (2011). ER, PgR, HER-2, Ki-67, topoisomerase IIalpha, and nm23-H1 proteins expression as predictors of pathological complete response to neoadjuvant chemotherapy for locally advanced breast cancer. Med Oncol.

[9] Prisack HB, Karreman C, Modlich O (2005). Predictive biological markers for response of invasive breast cancer to anthracycline/cyclophosphamide-based primary (radio-)chemotherapy. Anticancer Res.

[10] Zhou B, Yang DQ, Xie F (2008). Biological markers as predictive factors of response to neoadjuvant taxanes and anthracycline chemotherapy in breast carcinoma. Chin Med J.

[11] Burcombe RJ, Makris A, Richman PI (2005). Evaluation of ER, PgR, HER-2 and Ki-67 as predictors of response to neoadjuvant anthracycline chemotherapy for operable breast cancer. Br J Cancer.

[12] Hirata T, Shimizu C, Yonemori K (2009). Change in the hormone receptor status following administration of neoadjuvant chemotherapy and its impact on the long-term outcome in patients with primary breast cancer. Br J Cancer.

[13] Tacca O, Penault-Llorca F, Abrial C (2007). Changes in and prognostic value of hormone receptor status in a series of operable breast cancer patients treated with neoadjuvant chemotherapy. Oncologist.

[14] Zhang N, Moran MS, Huo Q (2011). The hormonal receptor status in breast cancer can be altered by neoadjuvant chemotherapy: a meta-analysis. Cancer Investig.

[15] Huober J, von Minckwitz G, Denkert C (2010). Effect of neoadjuvant anthracycline-taxane-based chemotherapy in different biological breast cancer phenotypes: overall results from the GeparTrio study. Breast Cancer Res Treat.

[16] Kim SI, Sohn J, Koo JS, Park SH, Park HS, Park BW (2010). Molecular subtypes and tumor response to neoadjuvant chemotherapy in patients with locally advanced breast cancer. Oncology.

[17] Shet T, Agrawal A, Chinoy R, Havaldar R, Parmar V, Badwe R (2007). Changes in the tumor grade and biological markers in locally advanced breast cancer after chemotherapy—implications for a pathologist. Breast J.

[18] Chen S, Chen CM, Yu KD, Zhou RJ, Shao ZM (2012). Prognostic value of a positive-to-negative change in hormone receptor status after neoadjuvant chemotherapy in patients with hormone receptor-positive breast cancer. Ann Surg Oncol.

[19] Parinyanitikul N, Lei X, Chavez-MacGregor M, et al. Receptor status change from primary to residual breast cancer after neoadjuvant chemotherapy and analysis of survival outcomes. Clin Breast Cancer 2015; 15:153-160. 10.1016/j.clbc.2014.09.006 25454687

[20] Niikura N, Tomotaki A, Miyata H (2016). Changes in tumor expression of HER2 and hormone receptors status after neoadjuvant chemotherapy in 21,755 patients from the Japanese breast cancer registry. Ann Oncol.

[21] Matsubara N, Mukai H, Masumoto M (2014). Survival outcome and reduction rate of Ki-67 between pre- and post-neoadjuvant chemotherapy in breast cancer patients with non-pCR. Breast Cancer Res Treat.

[22] Matsubara N, Mukai H, Fujii S, Wada N (2013). Different prognostic significance of Ki-67 change between pre- and post-neoadjuvant chemotherapy in various subtypes of breast cancer. Breast Cancer Res Treat.

